# Multi-Stability and Consequent Phenotypic Plasticity in AMPK-Akt Double Negative Feedback Loop in Cancer Cells

**DOI:** 10.3390/jcm10030472

**Published:** 2021-01-26

**Authors:** Adithya Chedere, Kishore Hari, Saurav Kumar, Annapoorni Rangarajan, Mohit Kumar Jolly

**Affiliations:** 1 Department of Molecular Reproduction, Development, and Genetics, Indian Institute of Science, Bangalore 560012, India; adithyac@iisc.ac.in (A.C.); skumar2@fredhutch.org (S.K.); 2 Centre for BioSystems Science and Engineering, Indian Institute of Science, Bangalore 560012, India; kishorehari@iisc.ac.in; 3 Basic Sciences Division, Fred Hutchinson Cancer Research Center, Seattle, WA 98109, USA

**Keywords:** phenotypic plasticity, bistability, double negative feedback loop, AMPK, Akt, matrix deprivation, anchorage independence

## Abstract

Adaptation and survival of cancer cells to various stress and growth factor conditions is crucial for successful metastasis. A double-negative feedback loop between two serine/threonine kinases AMPK (AMP-activated protein kinase) and Akt can regulate the adaptation of breast cancer cells to matrix-deprivation stress. This feedback loop can significantly generate two phenotypes or cell states: matrix detachment-triggered pAMPK^high^/ pAkt^low^ state, and matrix (re)attachment-triggered pAkt^high^/ pAMPK^low^ state. However, whether these two cell states can exhibit phenotypic plasticity and heterogeneity in a given cell population, i.e., whether they can co-exist and undergo spontaneous switching to generate the other subpopulation, remains unclear. Here, we develop a mechanism-based mathematical model that captures the set of experimentally reported interactions among AMPK and Akt. Our simulations suggest that the AMPK-Akt feedback loop can give rise to two co-existing phenotypes (pAkt^high^/ pAMPK^low^ and pAMPK^high^/pAkt^low^) in specific parameter regimes. Next, to test the model predictions, we segregated these two subpopulations in MDA-MB-231 cells and observed that each of them was capable of switching to another in adherent conditions. Finally, the predicted trends are supported by clinical data analysis of The Cancer Genome Atlas (TCGA) breast cancer and pan-cancer cohorts that revealed negatively correlated pAMPK and pAkt protein levels. Overall, our integrated computational-experimental approach unravels that AMPK-Akt feedback loop can generate multi-stability and drive phenotypic switching and heterogeneity in a cancer cell population.

## 1. Introduction

Despite major advances in cancer research, metastasis remains clinically unsolved and claims the vast majority of cancer-related deaths. Metastasis is a highly inefficient process with extremely high (>99.8%) rates of attrition. A hallmark of cancer cells that can successfully metastasize is their ability to dynamically adapt to their changing microenvironments, called phenotypic plasticity or switching [1,2]. Thus, understanding the rules of phenotypic plasticity and identifying therapeutic perturbations to reduce the fitness of metastasizing cells can be crucial for restricting the disease aggressiveness.

Metastasizing cells can often display phenotypic plasticity along multiple interconnected axes. Two of the most well-characterized axes are epithelial-mesenchymal plasticity (EMP) and cancer stem cell (CSC) plasticity [3,4]. More recently, the axes of metabolic plasticity (i.e., switching between more glycolytic vs. more oxidative phosphorylating states) [5,6] and drug resistance (i.e., switching between drug-resistant and drug-sensitive states) [7,8] are being investigated. A hallmark of networks involved in plasticity along these axes is the presence of double negative or mutually inhibitory feedback loops that can generate two (or more) phenotypes that cells can acquire. In the context of EMP, ZEB1 forms such loops with miR-200 and GRHL2, thus giving rise to multiple states–epithelial (high miR-200 and GRHL2, low ZEB), mesenchymal (low miR-200 and GRHL2, high ZEB), and hybrid epithelial/ mesenchymal (medium levels of miR-200, GRHL2 and ZEB) [9]. Similarly, for CSC plasticity, LIN28 and let-7 inhibit each other [10], and for metabolic plasticity, HIF-1α and AMPK (AMP-activated protein kinase) can inhibit each other [5]. The emergent dynamics of the above mentioned loops has been thoroughly investigated, and they have been shown as capable of exhibiting multi-stability (i.e., co-existence of multiple phenotypes). Such multi-stability has been posited to underlie phenotypic switching; disrupting such loops can restrict phenotypic switching, as witnessed for feedback loops of ZEB1 with GRHL2 and miR-200 [11,12].

Recent work from our laboratory has uncovered a double-negative feedback loop between two serine/threonine kinases AMPK and Akt operating in the adaptation of breast cancer cells to matrix-deprivation [13]. In epithelial cells, matrix-deprivation usually drives programmed cell death known as “anoikis” [14], but few detached epithelial cells can develop resistance to anoikis [15,16]. AMPK (AMP-activated protein kinase) is activated in cells facing bioenergetic or metabolic stress and can switch on energy-generating catabolic processes such as glycolysis and inhibit energy-consuming anabolic processes such as lipid and protein synthesis [17]. Conversely, upon growth factor stimulation, Akt becomes activated, promoting anabolic processes of lipid and protein synthesis, driving cell growth and proliferation [18]. Upon matrix deprivation, AMPK is activated which drives upregulation of PHLPP2 protein levels which can inactivate Akt. On the other hand, upon matrix (re)attachment, Akt is activated which can repress AMPK activity through PP2Cα [13]. Thus, while adherent cells showed a (re)attachment-triggered pAkt^high^/pAMPK^low^ state, matrix-deprived cells demonstrated a detachment-triggered pAMPK^high^/pAkt^low^ state. However, because this analysis was done at a population (or bulk) level, two questions remain to be answered: (a) can these cell states/phenotypes co-exist in the same cell population?, and (b) can these two subpopulations ‘spontaneously’ switch between themselves to give rise to one another?

Here, we adopt an integrative computational-experimental approach to answer these questions. First, we develop a mechanism-based mathematical model that captures the set of experimentally reported interactions among AMPK, Akt, PHLPP2 and PP2Cα. Simulations reveal that the AMPK–Akt feedback loop can give rise to two phenotypes–pAkt^high^/ pAMPK^low^, and pAMPK^high^/pAkt^low^–that can co-exist in specific parameter regimes, and switch between one another under the influence of biological noise. Next, we segregated the two subpopulations in MDA-MB-231 cells and observed that, under adherent conditions, each of them was capable of giving rise to another, thus validating our model prediction. Finally, clinical data analysis revealed a negative correlation between pAMPK and pAkt protein levels in The Cancer Genome Atlas (TCGA) breast cancer and pan-cancer cohorts. Overall, our results suggest that the AMPK–Akt feedback loop can be bistable, and therefore drive phenotypic switching and non-genetic heterogeneity in a cancer cell population.

## 2. Materials and Methods

### 2.1. ODE Model of The AMPK-Akt Network

The dynamics of the species in the regulatory network (Figure 2A) are represented using a system of Ordinary differential equations (ODEs) given below:

(1) $$\frac{d}{dt}\left[AMPK\right]\;\;=\;k_{ac_{AMPK\;}}\times \left(total_{AMPK}-\;\left[AMPK\right]\right)-k_{dac_{AMPK}}\times H\left(n_{PP2Ca},\lambda_{PP2ca},PP2ca^{0},PP2c\alpha \right)\times \left[AMPK\right])$$

(2) $$\frac{d}{dt}\left[AKT\right]\;=\;k_{ac_{AKT}}\;\times \;\left(total_{AKT}-\;\left[AKT\right]\right)-k_{dac_{AKT}}\;\times \;H\left(n_{PHLPP2},\lambda_{PHLPP2},PHLPP2^{0},PHLPP2\right)\;\times \;\left[AKT\right])$$

(3) $$\frac{d}{dt}\left[PHLPP2\right]\;=\;k_{ac_{PHLPP2}}\;\times \;\left(total_{PHLPP2}-\;\left[PHLPP2\right]\right)\;\times \;H\left(n_{AMPK},\lambda_{AMPK},AMPK^{0},AMPK\right)-k_{dac_{PHLPP2}}\;\times \;\left[PHLPP2\right])$$

(4) $$\frac{d}{dt}\left[PP2Ca\right]\;=\;k_{ac_{PP2Ca}}\;\times \;\left(total_{PP2Ca}-\;\left[PP2Ca\right]\right)\;\times \;H\left(n_{AKT},\lambda_{AKT},AKT^{0},AKT\right)-k_{dac_{PP2Ca}}\;\times \;\left[PP2Ca\right])$$

Here, each equation represents the rate of change of the active levels of the entity given in the left-hand side of the equation. The total levels of the entities are constant and only switch between active and inactive states. The activation and deactivation rates of the species, given as k_ac(X)_ and k_dac(X)_, (X ∈{AMPK,AKT,PHLPP2,PPCA}), respectively, are taken in units of t^-1^; all other parameters are in arbitrary units. The regulatory interactions are represented using shifted hill function form [19] as given below:

(5) $$H\left(n,\;\lambda ,A^{0},A\right)\;=\;\frac{\left(1+\;\lambda \times \;{\left(\frac{A}{A^{0}}\right)}^{n}\right)}{\left(1+{\left(\frac{A}{A^{0}}\right)}^{n}\right)}$$

where A is the effector species, *n* is the Hill’s coefficient that represents the non-linearity of the regulatory interaction, λ is the maximum fold change in activation/deactivation rate caused by A. λ > 1 indicates activation and λ < 1 indicates inhibition, A^0^ is the Activation/Inhibition threshold level of A. The parameter value ranges, with the corresponding dimensions, are given in Table 1 and Table S1. Assumptions made in the model are given below:

- Total levels of each molecule are taken as a constant value of 100 arbitrary units (A.U.) and do not change throughout the simulation. However, the concentration of active and inactive molecular species can change.
- Only the active state of the molecule affects another molecule’s conversions between its active and inactive forms.
- Each molecule has its intrinsic activation and deactivation rate. The influence of interaction with other protein causing state changes is accounted by multiplying the corresponding rate term with a hill function.

### 2.2. Temporal Profiles and Steady State Estimation

10,000 Parameter sets were randomly sampled from the ranges mentioned in Table S1. For each parameter set, the ODE system was simulated using 1000 randomly generated initial conditions between the range (0–total molecules). The temporal profiles for each initial condition were computed numerically using the ode23 function of Matlab *R2019A* until 1000 time-steps. Initial conditions across distinct parameter sets reach steady state values by 1000 time-steps (Figure S1), thus the final state reported after 1000 time steps was considered to be the steady state value. For each parameter set, unique steady states of the system are taken from 1000 temporal profiles. Z-score calculation was performed across all the parameter sets for all the molecules. Based on the Z-score values of AMPK and Akt, parameter sets are grouped into four categories (LL, LH, HL, HH). Positive Z-score is considered as high state (H) and negative Z-score is considered as low state (L). A heatmap was generated based on the Z-scores obtained for all parameter sets using the ComplexHeatmap package in R [20].

### 2.3. Nullcline, Bifurcation and Phase Plane Analysis

For a given parameter set for the ODE system, nullclines for AMPK and Akt were obtained by setting their respective rates of change to zero and thus using constant value for the active level of that species. The corresponding steady state of the other three variables was obtained by temporal simulations of the modified ODE system as described above. Bifurcation analysis was done using the software package MATCONT [21]. Phase planes were obtained by combining multiple bifurcation diagrams.

### 2.4. Noise Induction

We used a simple formalism to induce noise which introduces a white noise sampled from a normal random distribution of mean 0 and variance *η*, *η* ∈ {20,30,40}. This noise is added to the species levels at fixed intervals (t_step_ = 100) in the simulation, representing the additive stochasticity to the species levels in the time period of simulation (t_total_ = 5000). The fixed time interval is based on the observation that the mean time taken by different initial conditions across diverse parameter sets to attain equilibrium is around 100 timesteps (Figure S1B).

### 2.5. Clinical Data

Reverse Phase Protein Array (RPPA) dataset for TCGA- Pan Cancer 32, Breast Cancer and Sarcoma were downloaded from https://www.tcpaportal.org/tcpa/download.html. Pearson’s correlation analysis between phosphorylated AMPK (pT172) and phosphorylated AKT (pT308 and pS473) levels were calculated using cor.test() function from stats package and scatterplot was generated using plot() function in R 3.6.1.

### 2.6. Cell Line and Culture Condition, Fluorescence Activated Cell Sorting (FACS) Sorting and Analysis of The Plasticity

Breast cancer cell line MDA-MB-231 was procured from The American Type Culture Collection (ATCC) and validated subsequently by STR analysis. These cells were cultured in DMEM (Sigma-Aldrich) supplemented with 10% FBS (fetal bovine serum) containing penicillin and streptomycin, at 37 °C in 5% CO2 incubator. Cells were trypsinised and counted before seeding for every experiment. For FACS sorting purposes, MDA-MB-231 cells stably expressing EGR1promoter-TurboRFP (readout for the ERK activity; RFP: Red Fluorescent Protein) were sorted into high and low RFP cells after culturing on 90 mm tissue culture dish. These sorted cells were cultured in attached condition for the indicated time-points. Analysis of the high and low RFP sorted cells for their ability to attend the original heterogeneity was performed after the indicated time point of the culture in attached condition. Representative data were analysed using Summit software V5.2.1.12465 (Beckman Coulter, Miami, FL, USA).

### 2.7. Markov Chain Modelling and Simulations

To study the transition dynamics between the phenotypes observed in FACS data, the phenotypic transition is modelled as a Markov chain. In this formalism, a population of cells consists of 3 phenotypes, namely, the green phenotype (low EGR), red phenotype (high EGR) and black phenotype. Therefore, at any time t, the population is represented as:

(6) $$F_{t}=\left[f_{R_{t}},\;f_{G_{t}},f_{B_{t}}\right]$$

where f_Rt_ (f_Gt_, f_Bt_) is the fraction of the red (green, black) phenotype in the cell population at time t. Because the population size is large, these fractions are approximated to be the probabilities of a cell in the population showing the corresponding phenotype. The transition rates are defined as the conditional probabilities of a cell switching to a phenotype at time t+1 given its phenotype at time t. These transition rates build a transition matrix as follows:

(7) $$T_{m}=\;\begin{array}{ccc} P_{R|R} & P_{G|R} & P_{B|R}\\ P_{R|G} & P_{G|G} & P_{B|G}\\ P_{R|B} & P_{G|B} & P_{B|B} \end{array}$$

The transition matrix is assumed to be constant over time. Following the Markov chain property, the population at t+1 is obtained as follows:

(8) $$F_{t+1}=F_{t}\;\times \;T_{m}$$

The R package CellTrans is used to infer the transition matrix from the FACS data. Briefly, the package makes use of the above mentioned property of Markov chains and back calculates T_m_ using the information of F_t_ and F_(t+1)_ from the FACS data. More details of the methodology are provided in [22].

Using the transition matrix, we simulated the trajectories of sorted cell populations. In each instance of the simulations, we start with a sorted population of size 10,000 cells. At each time step, the transition of a given cell from its current phenotype to a new phenotype is decided using a uniform random number and the row of the transition matrix corresponding to the current phenotype of the cell. 1000 such simulations were performed to obtain a distribution of the population fractions of cell phenotypes at each time point.

## 3. Results

### 3.1. AMPK-Akt Feedback Loop Can Give Rise To Two States: pAkt^high^/ pAMPK^low^ and pAMPK^high^/pAkt^lo^

First, we gathered experimentally curated information about interconnections among AMPK and Akt. AMPK and Akt can antagonistically regulate common downstream effectors such as mTOR signaling and FOXO signaling through differential phosphorylation [23]. Moreover, they can affect the activation status of one another. For instance, AMPK activating agents such as AICAR and phenformin can reduce the phosphorylation of Akt [24]. Adiponectin-activated AMPK can dephosphorylate Akt by increasing the activity of protein phosphatase 2A (PP2A) through dephosphorylating PP2Ac at Tyr307 [25]. On the other hand, upon insulin treatment, Akt is activated and it phosphorylates Ser487 of the serine/threonine rich loop (ST loop) in AMPK-α1 subunit, thus reducing subsequent phosphorylation and LKB1- or CAMKKβ-dependent AMPK activation at Thr172. Also, GSK3, another substrate of Akt, can phosphorylate the AMPK-α1 subunit at Thr481 and Ser477, further inhibiting AMPK activation by Thr172 phosphorylation [26]. Thus, AMPK and Akt pathways seem to inhibit the activity of one another.

**Figure 1:**
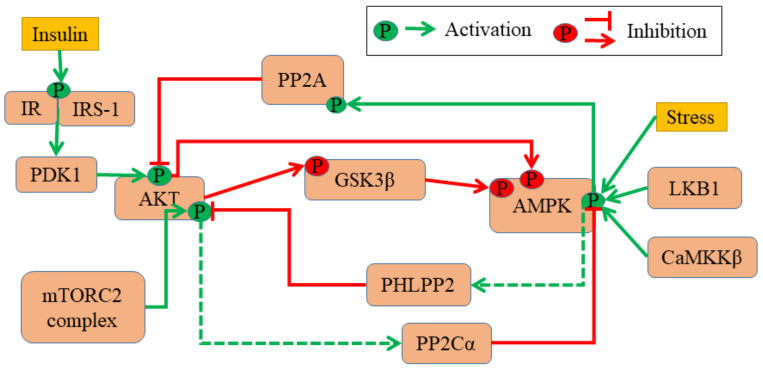
AMPK-Akt feedback loop. Regulatory network between AMPK and Akt. Green arrows and associated green ‘P’ circles denote activation by phosphorylation. Red arrows indicate deactivation by phosphorylation, and red hammerheads show deactivation by dephosphorylation. Solid lines show known molecular mechanisms, and dashed lines show scenarios where such information is not available.

**Figure 2:**
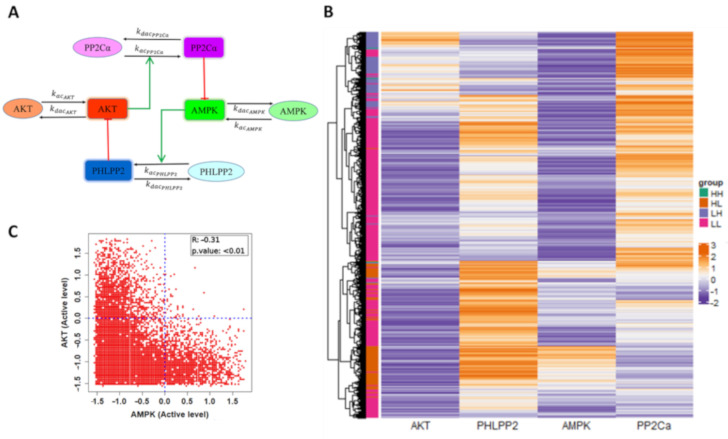
Dynamics of AMPK-Akt feedback loop. (**A**) Reduced network considered in the model containing a total of eight species – each of the four molecules (AMPK, Akt, PHLPP2, PP2Cα) has an active (rectangle) and an inactive (oval) form. Black arrows show the conversion of species from active to inactive and vice-versa. Red hammerheads represent inhibition, green arrows represent activation. Unless stated otherwise, further analysis shows the levels of only active species of the four molecules. (**B**) Heatmap of steady states attained by 10,000 random parameter sets generated from 1000 random initial conditions of the active levels of AMPK, Akt, PHLPP2, and PP2Cα. Color is based on the z-score calculated for the whole set of simulations across all parameter sets, orange represents positive z-score (high) and purple represents negative z-score (low). LL, HL, LH and HH denote the four states when considering the steady state levels of pAMPK, pAKT - pAMPK^low^/ pAkt^low^, pAMPK^high^/pAkt^low^, pAMPK^low^/ pAkt^high^ and pAMPK^high^/pAkt^high^. (**C**) Scatter plot of z-scores of AMPK and Akt steady state values represented in heatmap showing the state distribution. Pearson correlation coefficient, *p*-value are reported.

Such a double negative association between AMPK and Akt was also reported in breast cancer cells during matrix attachment and detachment. The activation of AMPK upon matrix-deprivation drove upregulation of PHLPP2 protein levels, which can inactivate Akt. On the other hand, when cells were (re)attached to the matrix, Akt was activated which repressed AMPK activity through PP2Cα [13] (Figure 1). Put together, the abovementioned interactions reveal a mutually inhibitory feedback loop between the AMPK and Akt. This loop is reminiscent of “toggle switches” formed by various ‘master regulators’ of two (or more) diverse cell states, seen during embryonic development and disease progression [27]. Such feedback loops can occur at transcriptional [28,29], post-transcriptional [30,31], and cell-cell communication levels [32,33]. Here, a ‘toggle switch’ is observed between two kinases. For further analysis, we have focused on interactions known in the context of matrix-deprivation in breast cancer cells (Figure 2A).

Next, we investigated the emergent dynamics of the AMPK-Akt feedback loop. For the sake of simplicity, we considered the set of interactions reported for breast cancer cells during matrix-deprivation (Figure 2A): (a) AMPK and Akt can switch back and forth between their phosphorylated (active) and dephosphorylated (inactive) forms, (b) phosphorylated AMPK (pAMPK) can upregulate the levels of PHLPP2 which promote the dephosphorylation of Akt, and (c) phosphorylated Akt (pAkt) can upregulate the levels of PP2Cα associated with AMPK, thus enhancing AMPK dephosphorylation. These interactions are represented via a set of four coupled ordinary differential equations (ODEs). Each ODE tracks temporal evolution of the levels of AMPK, Akt, PHLPP2, PP2Cα, and the set of ODEs is solved numerically to obtain the steady state values for each of these four variables. To identify the robust dynamic features of this set of experimentally identified interactions, the kinetic parameters were chosen from a biologically relevant range of values (Table 1; see Section 2 (Materials and Methods)). We chose 10,000 such unique parameter sets to represent the effects of cell-to-cell heterogeneity and 1000 initial conditions for each parameter set to characterize all the possible phenotypes across parameter sets.

We collated the levels of AMPK, Akt, PHLPP2 and PP2Cα obtained from all parameter combinations and plotted them using a heatmap. We observed the emergence of four major clusters – pAMPK^low^/pAkt^low^, pAMPK^high^/pAkt^low^, pAMPK^low^/pAkt^high^ and pAMPK^high^/pAkt^high^ state with pAMPK^high^/ pAkt^high^ state being the least frequent and pAMPK^low^/ pAkt^low^ state being the most frequent (Figure 2B and Figure S2). A scatter plot between the pAMPK and pAKT levels revealed a significantly negative correlation (Figure 2C, Figure S2), suggesting that pAMPK^high^/pAkt^low^ and pAMPK^low^/pAkt^high^ states can be the dominant outputs of the network. The other nodes of the network also showed expected correlations trends (Table S2). However, the model also suggests the possible existence of pAMPK^low^/pAkt^low^ and pAMPK^high^/ pAkt^high^ states; the biological evidence for their existence still remains inconclusive. The occurrence of these four states associated with respective ratios of activation and deactivation of AMPK and Akt as identified from parameter sampling. The pAMPK^low^/pAkt^low^ state was observed in parameter cases where the net activation rate of AMPK or Akt was quite low, independent of the AMPK or Akt activities (i.e., the effects of PHLPP2 or PP2Cα) (Figure S3). Relatively low levels of pAMPK and/or pAKT have been reported in certain experimental reports [34,35]. However, here in the context of breast cancer, we focused our attention to pAMPK^high^/ pAkt^low^ and pAMPK^low^/pAkt^high^ states [13], and the parameter sets that converged to them. In particular, we focused on parameter sets that enabled a co-existence of these two states (bistability).

### 3.2. The Two States (pAkt^high^/ pAMPK^low^ and pAMPK^high^/pAkt^low^) Can Co-Exist and Stochastically Switch Between One Another

To better understand bistability in this system, we performed nullcline and bifurcation analysis on the parameter sets showing bistability. First, for each parameter set, we constructed nullclines for pAMPK and pAKT. For a two-component system such as this, a nullcline represents the steady state levels of one component obtained over a range of values of the second component with its (i.e., the second component’s) rate of change over time being set to zero. The intersections of nullclines are, therefore, the points at which the rate of change for both the components is zero, i.e., a steady state of the system. The advantage of this approach over dynamical ODE-based simulations is that we can also identify unstable steady states that act as tipping points for transition from one stable steady state to another. In other words, for these parameter states, the two identified stable states can co-exist and can transition from one to another.

**Figure 3:**
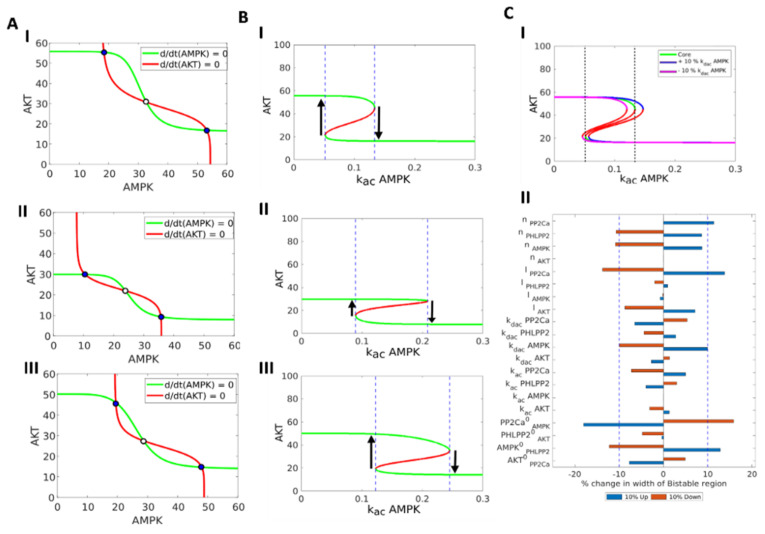
Nullcline, bifurcation and sensitivity analysis. (**A**) Nullclines for three representative bistable parameter sets (rows 1–3 in Table S3). Green curve is AMPK Nullcline (d/dt (AMPK)=0). Red curve is Akt Nullcline (d/dt (AKT)=0). Blue circles represent stable states, white circles represent unstable steady state. (**B**) Bifurcation of Akt steady state levels with respect to the activation rate of AMPK (k_ac AMPK) for three representative bistable parameter sets (rows 1–3 in Table S3). Green curves denote stable states and red curves denote unstable states. Region bound by the blue dashed line represents the bistable region, where both states can co-exist. (**C**) (I) Representative bifurcation of Akt levels with respect to activation rate of AMPK (k_ac AMPK), drawn for three different levels (control, ±10% change in deactivation rate of AMPK (k_dac AMPK). Green curve shows the control case, blue curve for +10% and magenta Curve for −10% of k_dac AMPK. (II) Sensitivity analysis of the width of the bistable region (length of the segment of the x axis between the black dotted lines in bifurcation diagram) for a parameter set (row #1 in Table S3) with changes in individual parameter by ±10% from the original value. Blue dotted line represents the ±10% change. Arrows show possible transitions between the different states.

Here, for a given parameter set, we first calculated the steady state levels of active Akt (pAKT) for different constant values of active AMPK (pAMPK) (green curve in Figure 3A(I). Next, we calculated the steady state levels of pAMPK for different fixed levels of pAkt (red curve in Figure 3A(I)). These two curves are called as nullclines and their intersections identify the steady states of the system, two of which are stable (filled circles), and one unstable (hollow circle). The two stable states are pAkt^high^/pAMPK^low^ and pAMPK^high^/pAkt^low^. The unstable state acts as a ‘tipping point’ beyond which a perturbation can allow switch from one state to another. Similar dynamical behavior was seen for other parametric combinations too, suggesting underlying bistability of the AMPK-Akt feedback loop (Figure 3A(II-III) and Figure S4).

To investigate how this feature of bistability may depend on various kinetic parameters in the model, we plotted a bifurcation diagram that tracks the levels of pAKT at varied values of activation rate of AMPK (k_ac_ AMPK). We chose k_ac_ AMPK as the bifurcation parameter to reflect the cases where the activation rate of AMPK can be altered by cell-intrinsic or cell-extrinsic factors. At low k_ac_ AMPK values (<0.05), pAkt levels are relatively higher, because the dephosphorylation of Akt by AMPK is weaker. Similarly, at high k_ac_ AMPK values (>0.13), pAkt levels are relatively lower, because of strong inactivation of Akt by AMPK. However, at intermediate range of these values (area bounded between dotted blue lines), a cell can exhibit bistability in terms of pAMPK levels, i.e., it can exist in either a pAkt^high^/pAMPK^low^ or pAMPK^high^/pAkt^low^ state (Figure 3B(I)). Similar trends are seen for other parameter sets shown earlier (Figure 3B(II-III)).

To quantify the range of bistability in the bifurcation diagram and its dependence on other kinetic parameters besides k_ac_ AMPK, we plotted this bifurcation at different values of activation rate of Akt (k_ac_ Akt) and observed that these curves (blue and magenta curves in Figure 3C(I) largely overlapped with that seen earlier (green curve in Figure 3C(I)). Next, we varied each parameter, one at a time, by +/− 10% and calculated the percentage change in the range of k_ac_ AMPK values enabling bistability, i.e., the distance between the dotted vertical lines. This sensitivity analysis suggested that the percentage change in this range was above 10% for only a few parameters such as those defining the effect of PP2Ca on AMPK (Figure 3C(II), Figures S5 and S6). These results underscore that the existence of bistability in AMPK-Akt feedback loop can be considered as largely robust to small parameter variations. Consequently, cells in an isogenic population may exist in two distinct states: pAkt^high^/pAMPK^low^ and pAMPK^high^/pAkt^low^.

**Figure 4:**
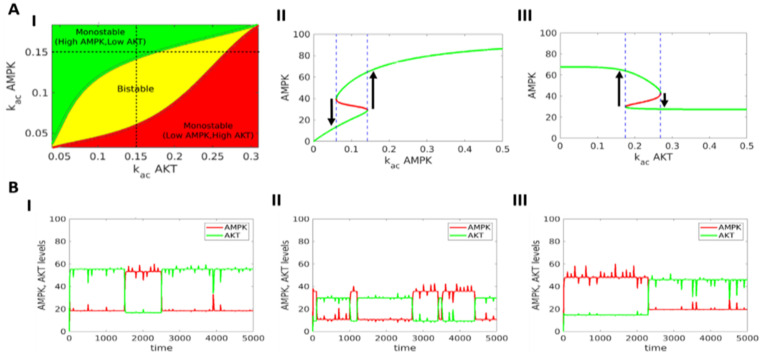
Phase plots and stochastic simulations. (**A**) (I) Phase diagram for two parameters –activation rates of AMPK and Akt – showing monostable and bistable regions. All other parameter values correspond to those given in row #1 in Table S3. (II) Bifurcation diagram of AMPK levels with respect to k_ac AMPK for a constant value of k_ac Akt = 0.15. (III) Same as (II) but with respect to k_ac Akt for a constant value of k_ac AMPK=0.15. Green curve shows stable states, red curve shows unstable states. Blue dotted lines show region of bistability. (**B**) Stochastic simulations showing trajectories of AMPK, Akt values under the influence of noise for three representative parameter sets (rows 1–3 in Table S3). Noise parameter value *η* = 20.

Next, we varied two parameters simultaneously, to map the two-dimensional parameter region in terms of (co-)existence of the two states. At high k_ac_ AMPK and low k_ac_ Akt, only the pAMPK^high^/pAkt^low^ state was observed. Similarly, low k_ac_ AMPK and high k_ac_ Akt allowed only the pAkt^high^/pAMPK^low^ state. Both these states co-existed in a parameter region lying between these extremes (yellow region in Figure 4A(I)). These trends were reinforced based on bifurcation diagrams drawn for an intermediate value of k_ac_ Akt (= 0.15), with k_ac_ AMPK as the bifurcation parameter. Bistability existed for intermediate values of k_ac_ AMPK; while higher or lower values led to monostable regions where only one state existed (Figure 4A(II)). Bifurcation diagram with k_ac_ Akt as the parameter confirmed the trend (Figure 4A(III)). Similar characteristics dynamics was seen for other parameter sets (Figure S7).

The co-existence of two (or more) states in a bifurcation diagram indicates that they may ‘spontaneously’ switch among one another. Thus, we performed stochastic simulations to examine for state switching under the influence of biological noise. These simulations revealed that cells can switch back and forth between the pAkt^high^/ pAMPK^low^ and pAMPK^high^/pAkt^low^ states for varying strengths of noise parameter (Figure 4B and Figure S8), highlighting that spontaneous state switching may be an outcome of the AMPK-Akt loop.

Such state switching implies that, when a cell population is sorted into subpopulations, it is possible for a subpopulation to give rise to another and potentially generate a population distribution similar to that seen in the parental population [36,37]. The rates of switching to and from a subpopulation to/from another one may be unequal, depending on the relative stability of the two (or more) states, evidenced by different mean residence times in a given state [38].

### 3.3. Experimental and Clinical Data Supports The Model Predictions of Bistability in AMPK-Akt Loop

**Figure 5:**
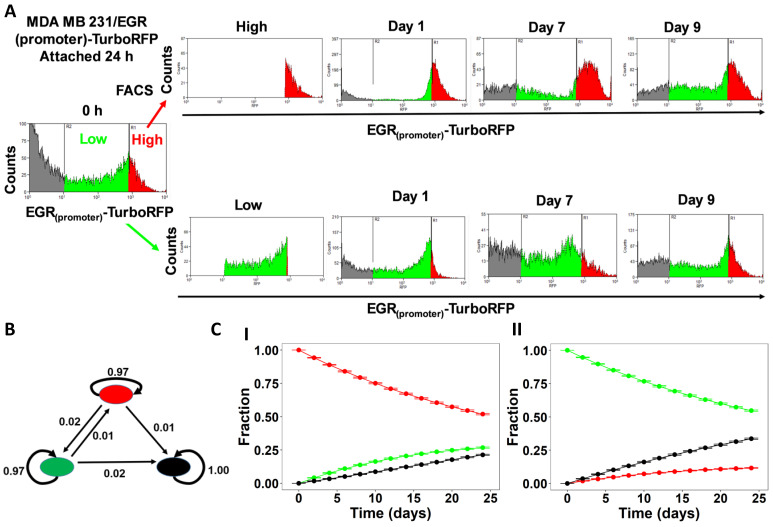
Stochastic state transitions. (**A**) Experimental validation of AMPK-Akt feedback loop using MDA-MB-231 EGR1-Turbo RFP cell lines sorted for high and low RFP expressing population using FACS. The RFP high population (red) corresponds to low pAMPK and high pAkt, and RFP^low^ (green) population corresponds to low EGR activity, high pAMPK, and low pAkt. Grey population correspond to cells that have lost the vector. Histograms show the population composition after 1, 7 and 9 days when started with distinct RFP^high^ (red, top panel) and RFP^low^ (green, bottom panel) populations. (**B**) State transition graph: each node is a cell phenotype colored the same as FACS data for representative purposes, and each edge represents transition between the corresponding phenotypes. Transition rates (per day) calculated from the Markov model are shown on the corresponding arrows. (**C**) Predicted evolution of population heterogeneity when started from the initial population fractions, equivalent to distinct populations in the FACS data, using the transition probabilities calculated here. Error bars represent mean ± standard deviation from 1000 such simulations.

To experimentally interrogate our observations of spontaneous state switching between pAkt^high^/pAMPK^low^ and pAMPK^high^/pAkt^low^ states, we performed FACS based cell sorting experiments in MDA-MB-231 cells stable for EGR1(promoter)-TurboRFP (EGR1 promoter-reporter system). In this approach, EGR1 promoter is used as a readout for AMPK activity, where high EGR activity (assessed by RFP intensity) corresponds to low AMPK activity and vice-versa [39]. Cells were grown in attached condition and sorted into high and low RFP group by FACS. These cells were cultured again for the indicated times in attached condition and we observed that these cells regained their original heterogeneous nature with time (Figure 5A and Figure S9A).

To quantify the observed phenotypic transitions between low and high AMPK (red and green populations respectively) cells, we constructed a discrete time Markov chain model [31]. The model assumes that transition rates between these cell types are independent of each other and do not change with time. We further assumed that the non-expressing cells (black population in the FACS distributions) do not transition into any other cell type. Using the R package CellTrans [22], we obtained the transition matrix, an ordered collection of transition rates (Figure 5B and Figure S9B). Using these transition rates, we simulated the evolution of population composition starting from homogeneous populations (Figure 5C and Figure S9C). The model predicts a higher transition rate from pAkt^high^/pAMPK^low^ to pAMPK^high^/pAkt^low^ phenotype, than that of pAMPK^high^/pAkt^low^ to pAkt^high^/pAMPK^low^, suggesting that the pAMPK^high^/pAkt^low^ population may be relatively more stable.

**Figure 6:**
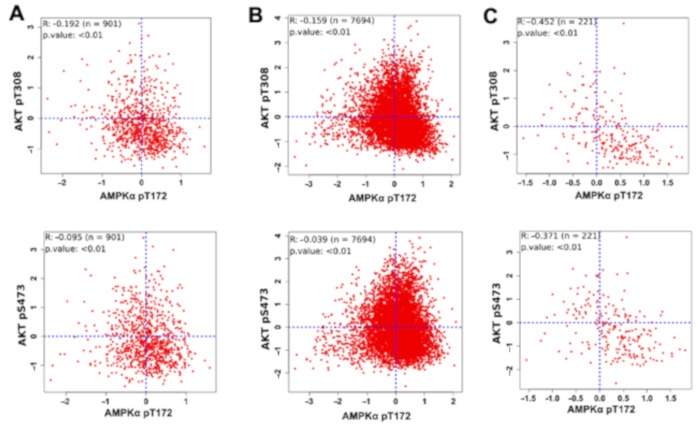
Clinical validation of AMPK-Akt double negative feedback loop. Scatter plot of active levels of AMPK (AMPK pT172) and Akt (pT308 and pS473) in (**A**) Breast cancer cohort (TCGA-BRCA) (*n* = 901), (**B**) Pan Cancer cohort of 32 cancer types (*n* = 7694) and (**C**) Sarcoma cohort (TCGA-SARC) (*n* = 221). Each dot represents one patient and coordinates correspond to the protein expression levels capture using RPPA (reverse phase protein array) with Akt and AMPK active forms (AMPK-pT172, Akt-pT308 and Akt-pS473). Pearson’s correlation coefficients and *p*-value are reported.

Further, to support our finding in the clinical setting, we performed a correlation of pAkt and pAMPK levels in patient samples using RPPA data from the TCGA cohort. A significant negative correlation was observed between pAkt and pAMPK levels across various cancer types (Figure 6, Table S4). This correlation was stronger for one of the two different phosphorylated versions of pAKT (compare top row vs. bottom row in Figure 6). These data further support the existence of bistability in the dynamics of AMPK-Akt feedback loop. Put together, our integrated computational-experiment analysis shows that the AMPK-Akt crosstalk can give rise to bistability and can lead to switching of states, driving heterogeneity at population level.

## 4. Discussion

Phenotypic switching can play crucial roles during cancer progression, as seen across cancer types. Prostate cancer cells can switch to a neuroendocrine-like state that is refractory to various therapies [40]. Similarly, in small cell lung cancer, cells under therapy-induced stress can reversibly switch to a hybrid neuroendocrine/mesenchymal state [41]. Besides these specific examples, EMP and CSCs are archetypal examples of phenotypic switching reported in many carcinomas [42,43] and non-epithelial tumors [44,45,46,47]. Recent progress in collecting high-throughput spatiotemporal data and mapping the regulatory networks underlying these axes of plasticity has led to developing complex mechanism-based and population-based mathematical models to decode dynamical traits of phenotypic switching such as dose-dependence, reversibility, hysteresis and transition rates among the cell states [48,49,50,51,52,53,54,55,56].

A hallmark of regulatory networks enabling phenotypic switching in cancer cell populations is multi-stability, i.e., the ability of isogenic cells to reversibly acquire diverse phenotypes [5,11,12,57,58,59,60,61], as reported earlier also for bacterial [62] and viral [63] populations. Multi-stability can enable ‘spontaneous’ switching among cell phenotypes (different attractors in the Waddington’s landscape) due to biological noise (that can operate at multiple levels including transcriptional or conformational [64,65]), and thus facilitate non-genetic heterogeneity [8,66]. For instance, in the context of EMP, PMC42-LA cells showed a bimodal distribution of EpCAM, and either subpopulation (EpCAM-^high^ or EpCAM-^low^) was capable of generating the other without any exogenous overt induction [53]. Similar observations have been reported in maintaining a dynamic equilibrium of CSCs and non-CSCs in breast cancer [67].

Here, we demonstrate breast cancer cells switching between pAkt^high^/pAMPK^low^ and pAMPK^high^/pAkt^low^ states in adherent cell populations. Together with reinforcing observations of switching among these subpopulations in matrix-detached conditions using the same reporter construct in MDA-MB-231 cells [39], our results strongly support the existence of multi-stability in AMPK-Akt double negative feedback loop, as predicted by our mechanism-based mathematical model. A limitation of our mathematical model is the limited set of interactions between AMPK and Akt that we incorporated for the purpose of characterizing their dynamics in the context of matrix-deprivation. Other context-dependent interactions between AMPK and Akt have been reported, for instance, AMPK can activate Akt in acute lymphoblastic leukemia [68]. Also, AMPK mediated phosphorylation of Skp2 at S256 can activate the Skp2 SCF complex, driving K63-linked ubiquitination and eventual activation of Akt [69]. However, these interactions have not been yet observed in matrix-deprivation conditions. Such context-specific differences may allow for other dynamics for AMPK and Akt, such as oscillations seen in MCF10A cells upon inhibition of glycolysis and mitochondrial ATPase [70]. Intriguingly, AMPK can form double negative feedback loops with other molecules such as mTORC1 [71], which is involved in a similar feedback loop with ULK1 [72]. Coupling of such “toggle switches” can influence the emergence of multi-stability [73].

Another salient feature of multi-stability is hysteresis, as observed for bacterial cells [74] and for EMP in cancer cells [11]. Future experiments should investigate the possibility and implications of hysteresis in AMPK-Akt feedback loop driving the adaptation of breast cancer cells to matrix-deprivation (in other words, anchorage-independence) stress. Also, anchorage-independence has been shown to be associated with other axes of phenotypic plasticity: EMP [75,76,77], CSCs [78], and metabolic reprogramming [79,80,81]. However, a systems-level understanding of coordination of cell states along these interconnected axes remains elusive. Future integrative computational-experimental efforts, similar to the approach taken here, can be critical in investigating such coupled dynamics of phenotypic plasticity during the challenging metastatic cascade and identify therapeutic targets that can impact multiple axes of plasticity simultaneously.

## Figures and Tables

**Table 1:** Parameter values, their description and ranges used for random circuit perturbation simulation.

Parameter	Description	Value Range
total_AMPK_	Total level of AMPK	100
total_AKT_	Total level of AKT	100
total_PHLPP2_	Total level of PHLPP2	100
total_PP2Cα_	Total level of PP2Cα	100
k_ac_AMPK_	Activation rate of AMPK	(0.02–0.2)
k_ac_AKT_	Activation rate of AKT	(0.02–0.2)
k_ac_PHLPP2_	Activation rate of PHLPP2	(0.02–0.2)
k_ac_PP2Cα_	Activation rate of PP2Cα	(0.02–0.2)
k_dac_AMPK_	Deactivation rate of AMPK	(0.02–0.2)
k_dac_AKT_	Deactivation rate of AKT	(0.02–0.2)
k_dac_PHLPP2_	Deactivation rate of PHLPP2	(0.02–0.2)
k_dac_PP2Cα_	Deactivation rate of PP2Cα	(0.02–0.2)
λ_PP2cα_	Effect of PP2Cα on AMPK	(5–10)
λ_PHLPP2_	Effect of PHLPP2 on AKT	(5–10)
λ_AKT_	Effect of AKT on PP2Cα	(5–10)
λ_AMPK_	Effect of AMPK on PHLPP2	(5–10)
*n*_PP2Cα_	Hill coefficient of PP2Cα for deactivation of AMPK	4, 5, 6
*n*_PHLPP2_	Hill coefficient of PHLPP2 for deactivation of AKT	4, 5, 6
*n*_AKT_	Hill coefficient of AKT for activation of PP2Cα	4, 5, 6
*n*_AMPK_	Hill coefficient of AMPK for activation of PHLPP2	4, 5, 6
PP2Cα^0^	Threshold value of PP2Cα for deactivation of AMPK	(0.25–0.75) × total_PP2Cα_
PHLPP2^0^	Threshold value of PHKLPP2 for deactivation of AKT	(0.25–0.75) × total_PHLPP2_
AMPK^0^	Threshold value of AMPK for activation of PHLPP2	(0.25–0.75) × total_AMPK_
AKT^0^	Threshold value of AKT for activation of PP2Cα	(0.25–0.75) × total_AKT_

AMPK: AMP-activated Protein Kinase, PHLPP2: Plectistin Homology Domain And Leucine Rich Repeat Protein Phosphatase 2, PP2Cα: Protein Phosphatase 2C alpha.

## Data Availability

The corresponding codes are available at https://github.com/csbBSSE/AmpkAkt. Reverse Phase Protein Array (RPPA) dataset for TCGA- Pan Cancer 32, Breast Cancer and Sarcoma were downloaded from https://www.tcpaportal.org/tcpa/download.html.

## Supplementary Materials

The following are available online at https://www.mdpi.com/2077-0383/10/3/472/s1, Table S1: Shows the literature reported values of parameter values. Table S2: Shows the Pearson’s correlation values and corresponding *p* values for the comparisons between AMPK, Akt, PHLPP2 and PP2Cα for three replicates, Table S3: Shows the 10 representative parameter sets used in the study, Table S4: Shows the Pearson’s correlation values and *p* values between AMPK (pT172) and Akt (pT308 and pS473) for 32 different TCGA cancer cohorts.
