# Supplementary material for: Multi-Stability and Consequent Phenotypic Plasticity in AMPK-Akt Double Negative Feedback Loop in Cancer Cells
Source: J Clin Med. 2021 Jan 26;10(3):472. doi: 10.3390/jcm10030472 (PMC7865639; doi:10.3390/jcm10030472)
Supplement: Supplementary file 1 [file jcm-10-00472-s001.zip › SI revised/07Jan_Supplementary_figures_AC.docx]

**
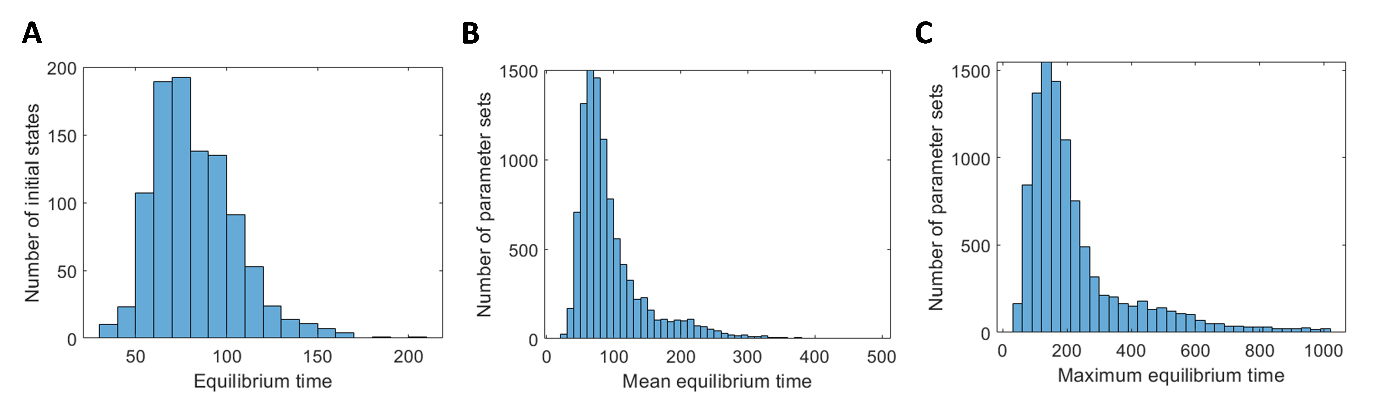
Figure S1: Equilibrium time distributions**

**A** Histogram of time taken to attained equilibrium states for 1000 random initial states for a representative parameter set

**B** Histogram of mean time taken to attained equilibrium states for 10000 random parameter sets with 1000 random initial states

**C** Histogram of maximum time taken to attained equilibrium states for 10000 random parameter sets with 1000 random initial states


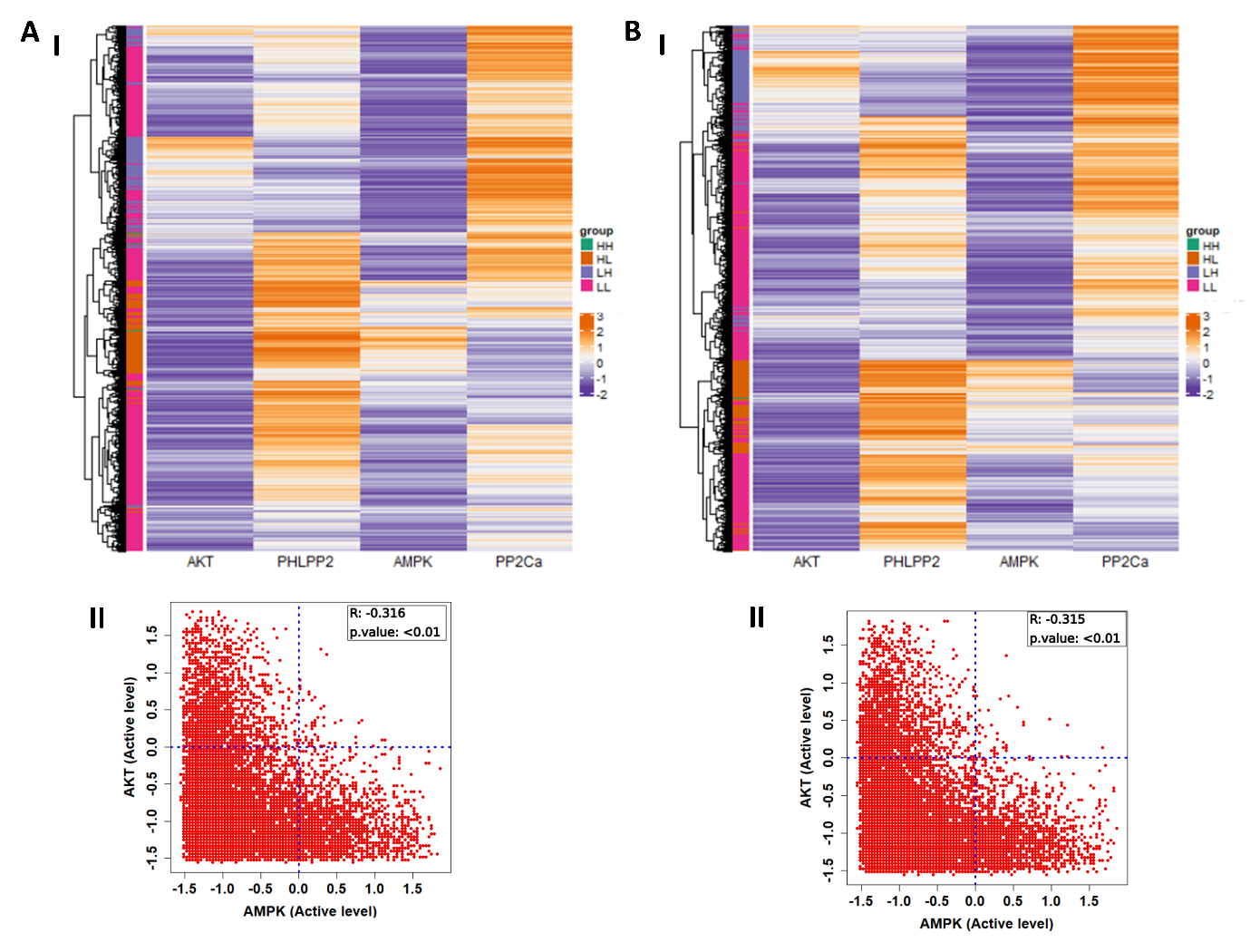
**Figure S2:**

**A-B (I)** Heatmap of final states attained by 10,000 random parameter sets generated from 1000 random initial states of the active levels of AMPK, AKT, PHLPP2, PP2Cα. Color range of the cells is based on z-score calculated for the whole set, orange represents positive z-score, and purple represents negative z-score. LL, HL, LH and HH denote the four states - pAMPK^low^/ pAkt^low^, pAMPK^high^/pAkt^low^, pAMPK^low^/ pAkt^high^ and pAMPK^high^/pAkt^high^.

**A-B (II)** Scatter plot of AMPK and AKT z-score values represented in the heatmap emphasising the distribution of states. Pearson correlation coefficient, p-value are reported. (A) for parameter set 2 and (B) for parameter set 3


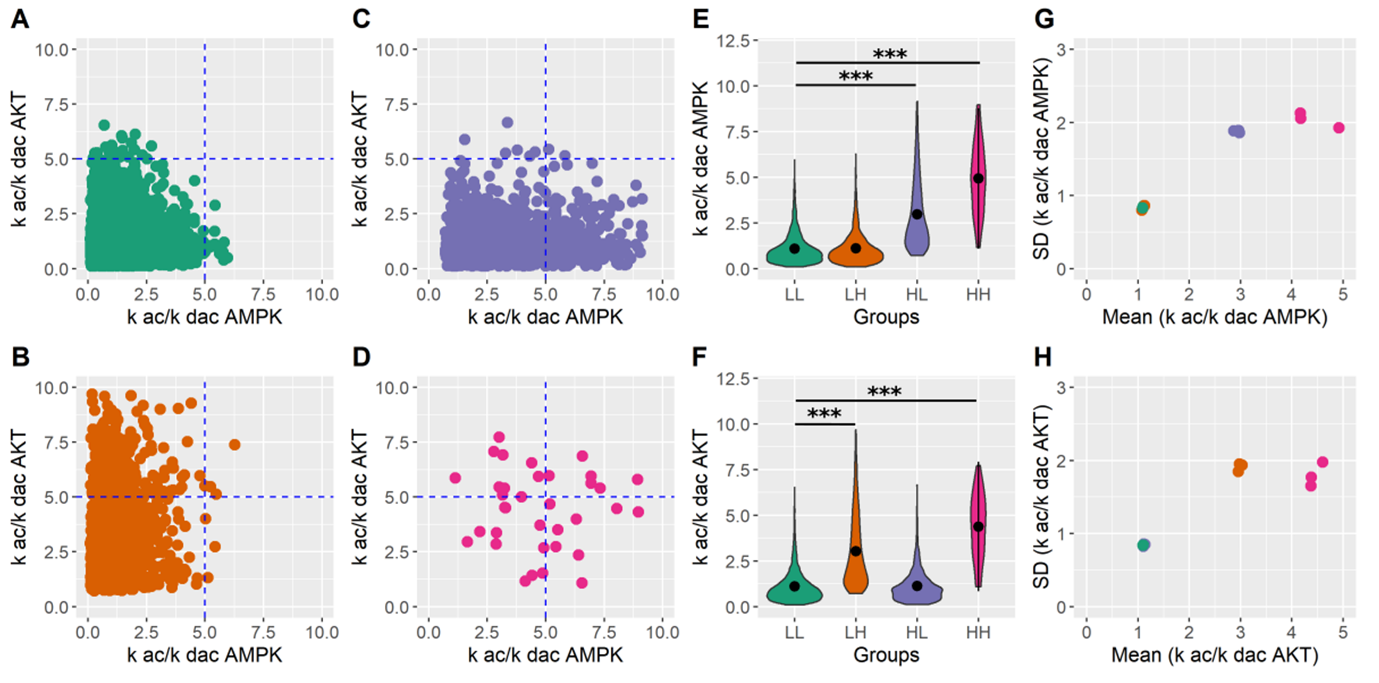


**Figure S3:**

**A-D** Scatter plot for k_ac/k_dac AMPK and Akt for four groups (LL, HL, LH and HH). **E, F** Violin plots showing the distribution k_ac/k_dac AMPK and Akt values across different groups. Black dot represents the mean of the distribution and *** denotes the p value <10^-5^. **G, H** Scatter plot for mean and standard deviation (SD) of k_ac/k_dac AMPK and Akt values across three replicates of simulations. Each dot represents the data from one replicate; panels A-F show the data for one replicate. LL, HL, LH and HH denote the four states - pAMPK^low^ / pAkt^low^, pAMPK^high^ /pAkt^low^, pAMPK^low^ / pAkt^high^ and pAMPK^high^ /pAkt^high^.


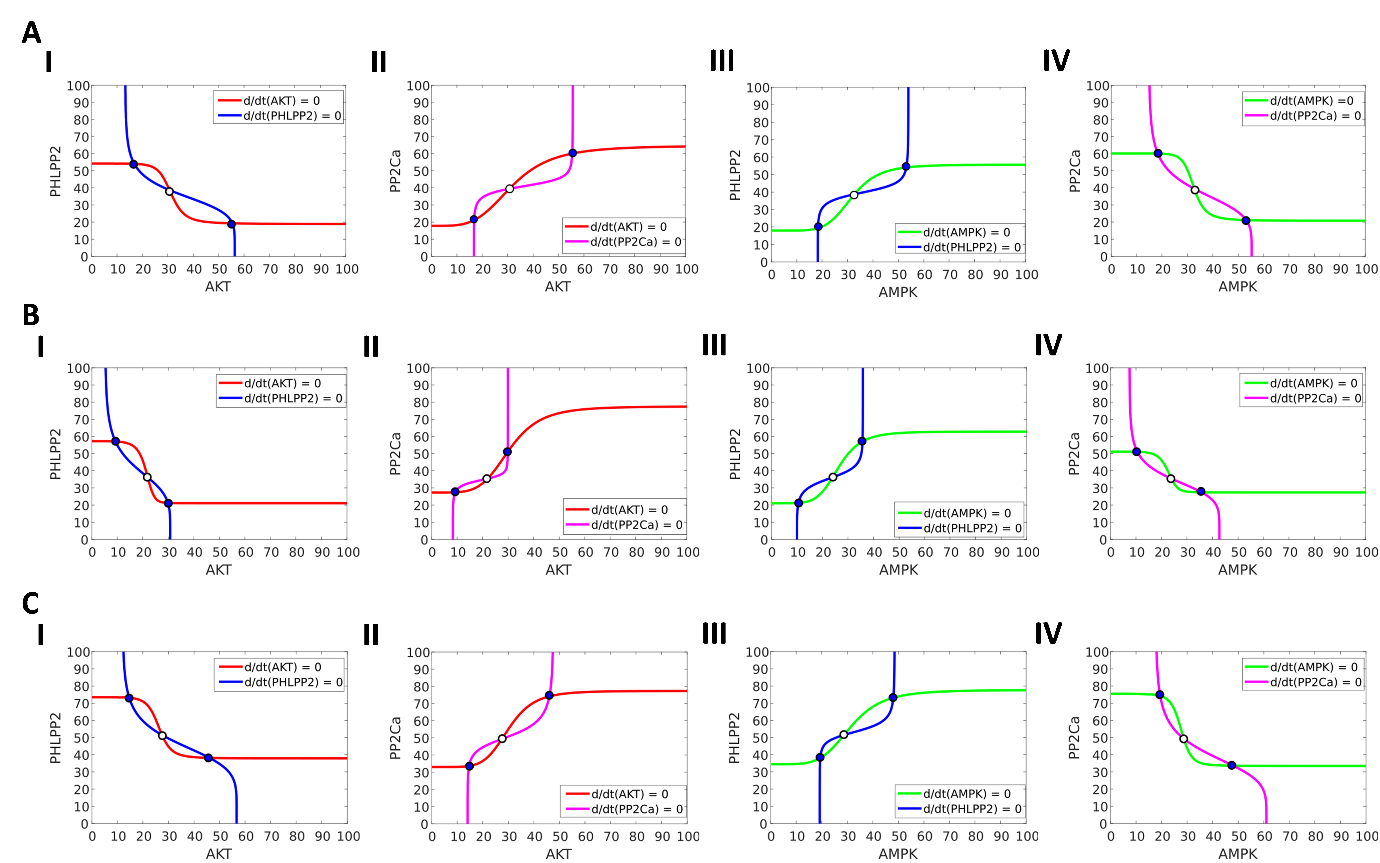


**Figure S4:**

Representative nullclines for three representative parameter sets. Red curve is Akt Nullcline [d/dt (AKT) =0]. Green curve is AMPK Nullcline [d/dt (AMPK) =0]. Blue curve is PHLPP2 Nullcline [d/dt (PHLPP2) =0]. Magenta curve is PP2Ca Nullcline [d/dt (PP2Ca) =0]. Blue circles represent stable states, white circles represent unstable steady state. A-C are for parameter sets 1-3 (row #1-3 in Table S3).


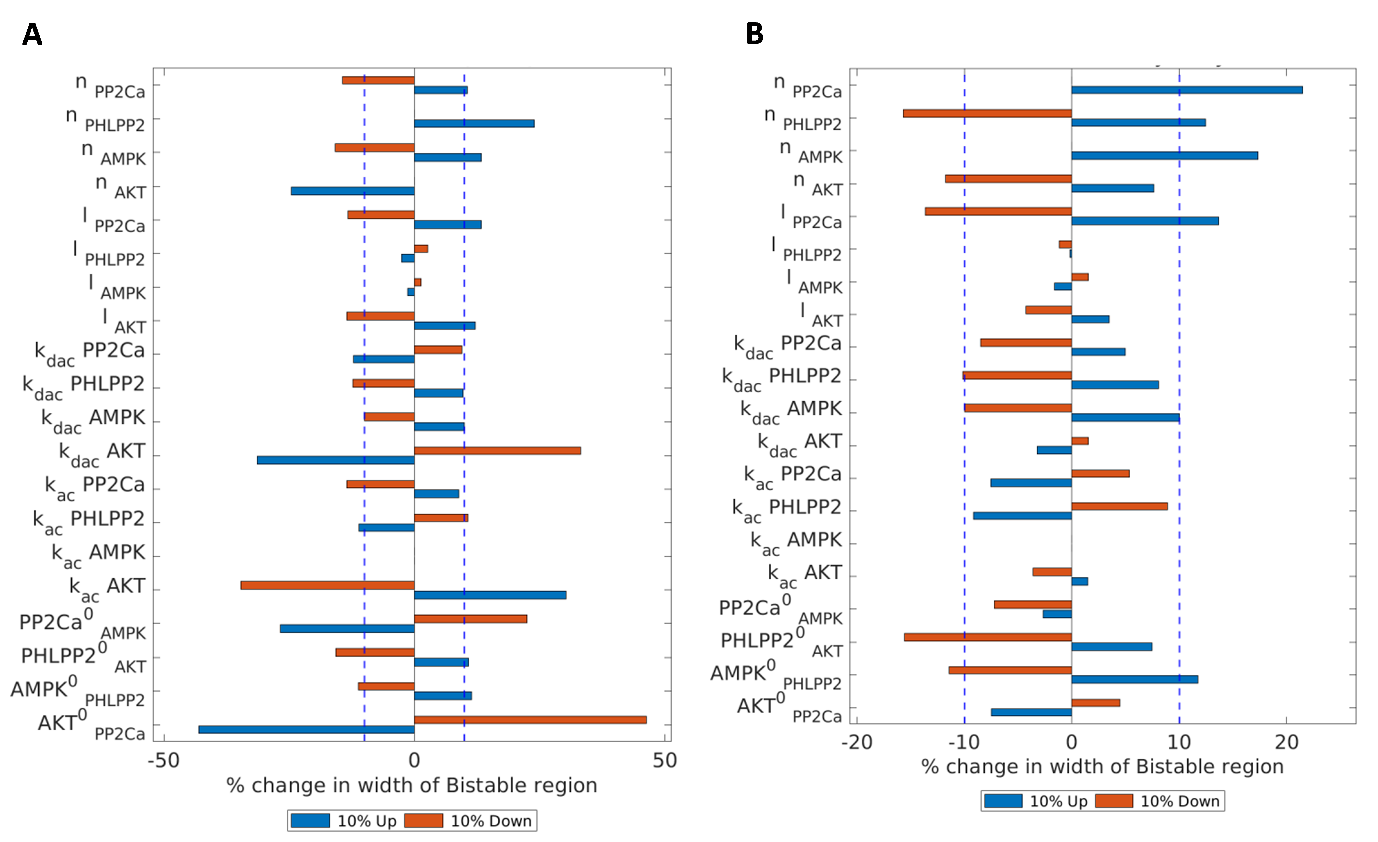
**Figure S5:**

Sensitivity of the width of the bistable region for a parameter set with changes in individual parameter by ±10% from the original value. Blue dotted line represents the ±10 % change. For parameter set 2-3 (rows #2-3 in Table S3)

**
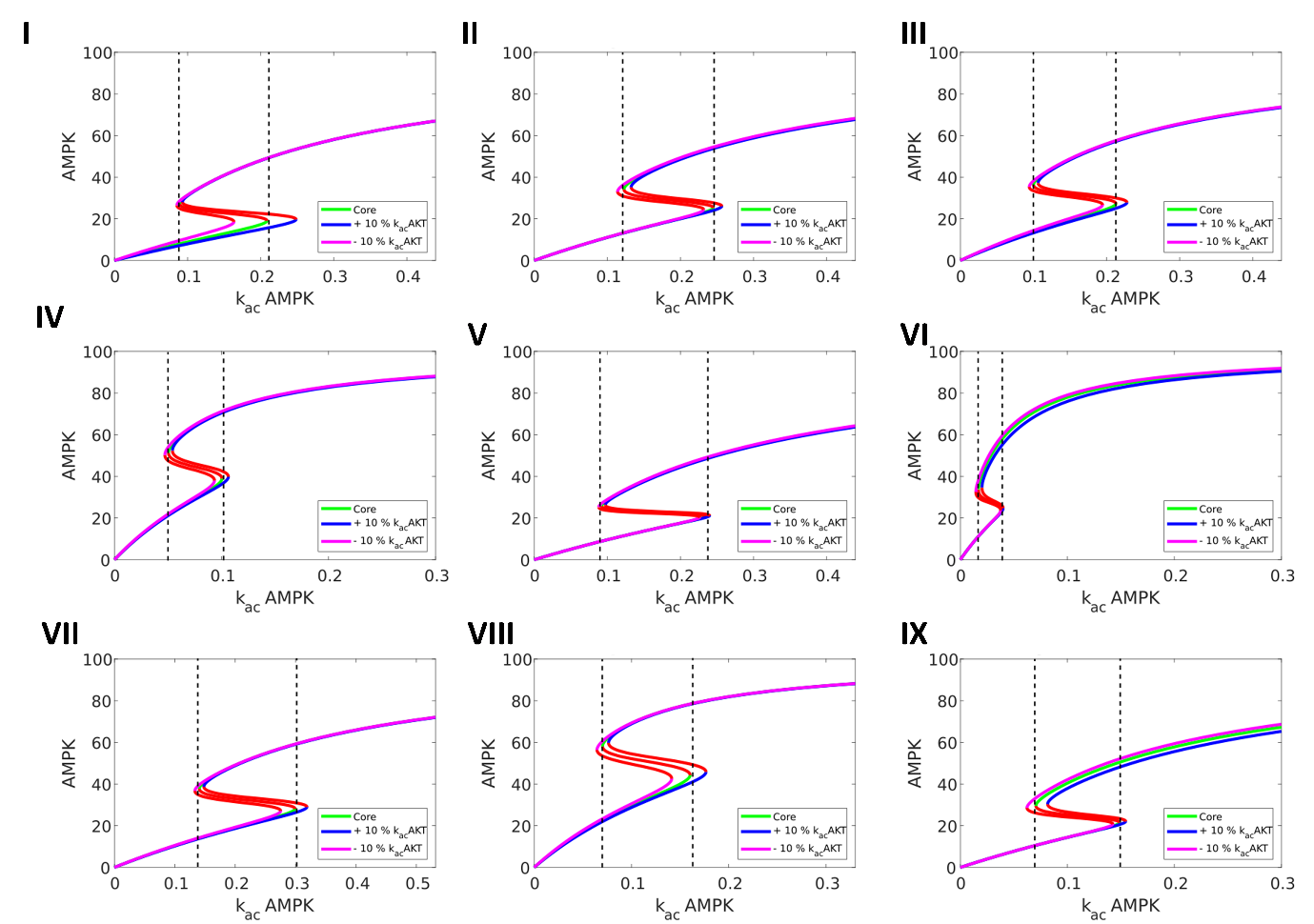
**

**Figure S6:**

Representative bifurcation of AMPK levels with respect to (±10% change) in the activation rate of AMPK (k_ac AMPK). Green curve is for the core value, blue curve for + 10 % and magenta curve for – 10 % of the activation rate of Akt (k_ac Akt). I – IX are for parameter sets 2-10 (row #2-10 in Table S3).


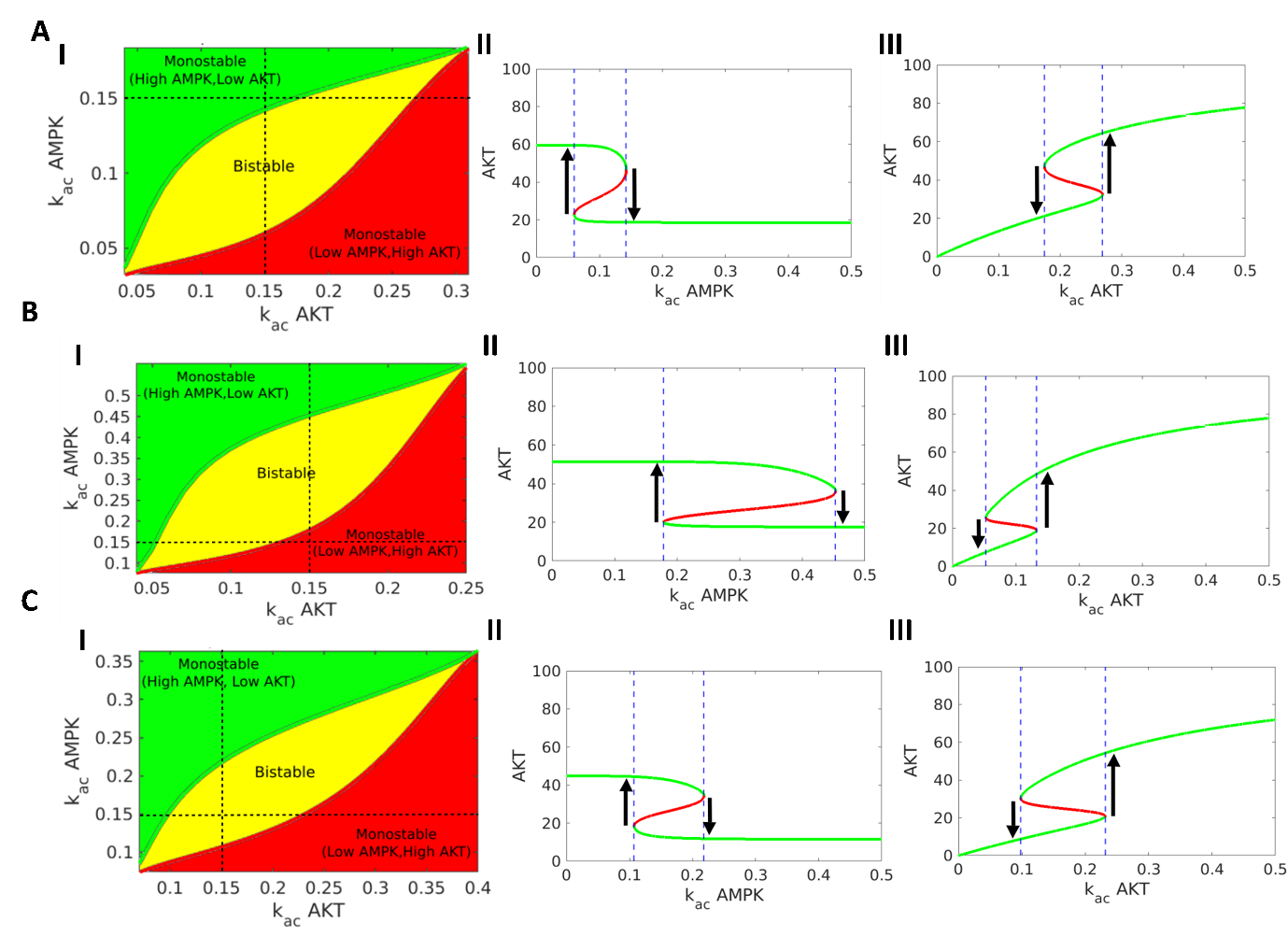


**Figure S7:**

(I) Phase diagram for two parameters –activation rates of AMPK and Akt – showing monostable and bistable regions. (II, III) Bifurcation of Akt levels with respect to k_ac Akt and k_ac AMPK under constant value (0.15) of k_ac AMPK and k_ac Akt, respectively. Green curve shows stable states, red curve shows unstable states. Blue dotted lines show region of bistability. (A- C) for parameter set 1- 3 ( rows #1-3 in Table S3).

**
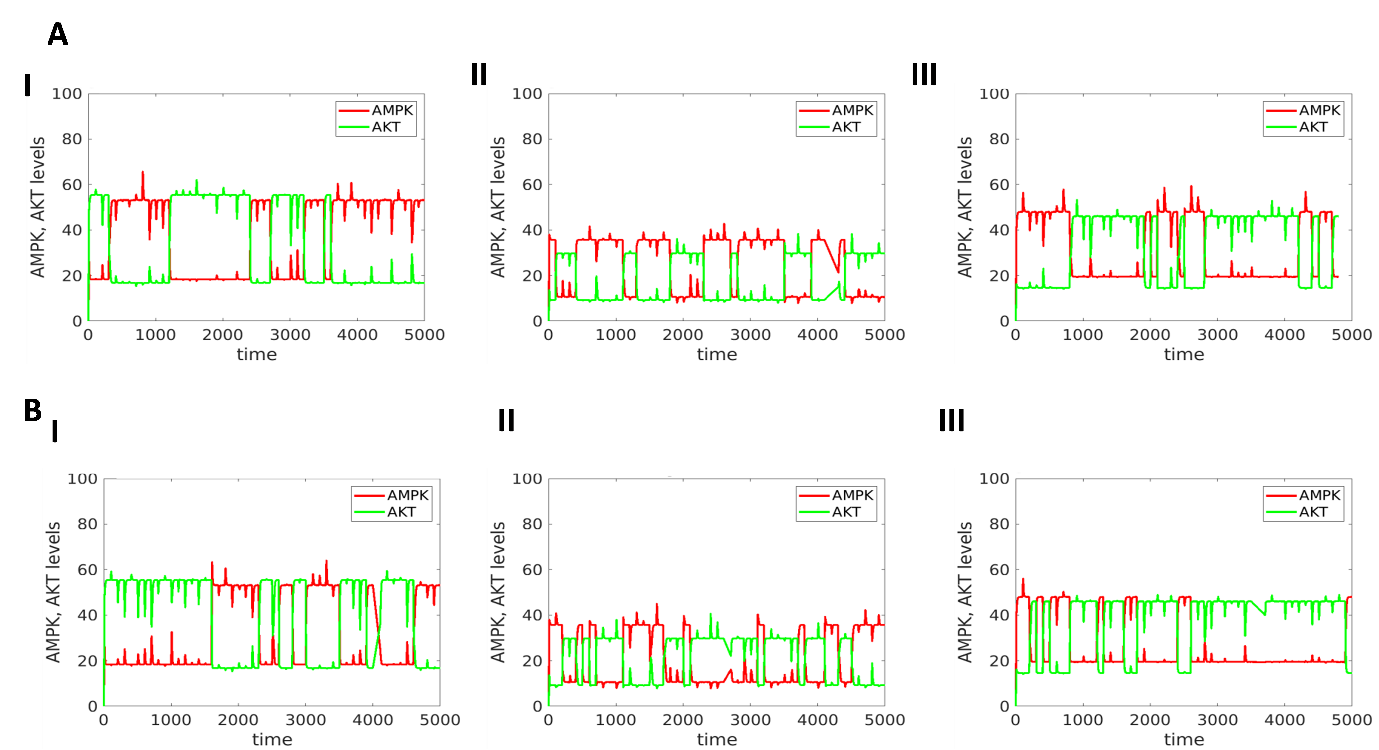
**

**Figure S8:**

Stochastic simulations showing trajectories of AMPK, Akt values under the influence of noise parameter **A** (η=3 0) and **B**(η =40) for three representative parameter sets (rows #1-3 in Table S3).


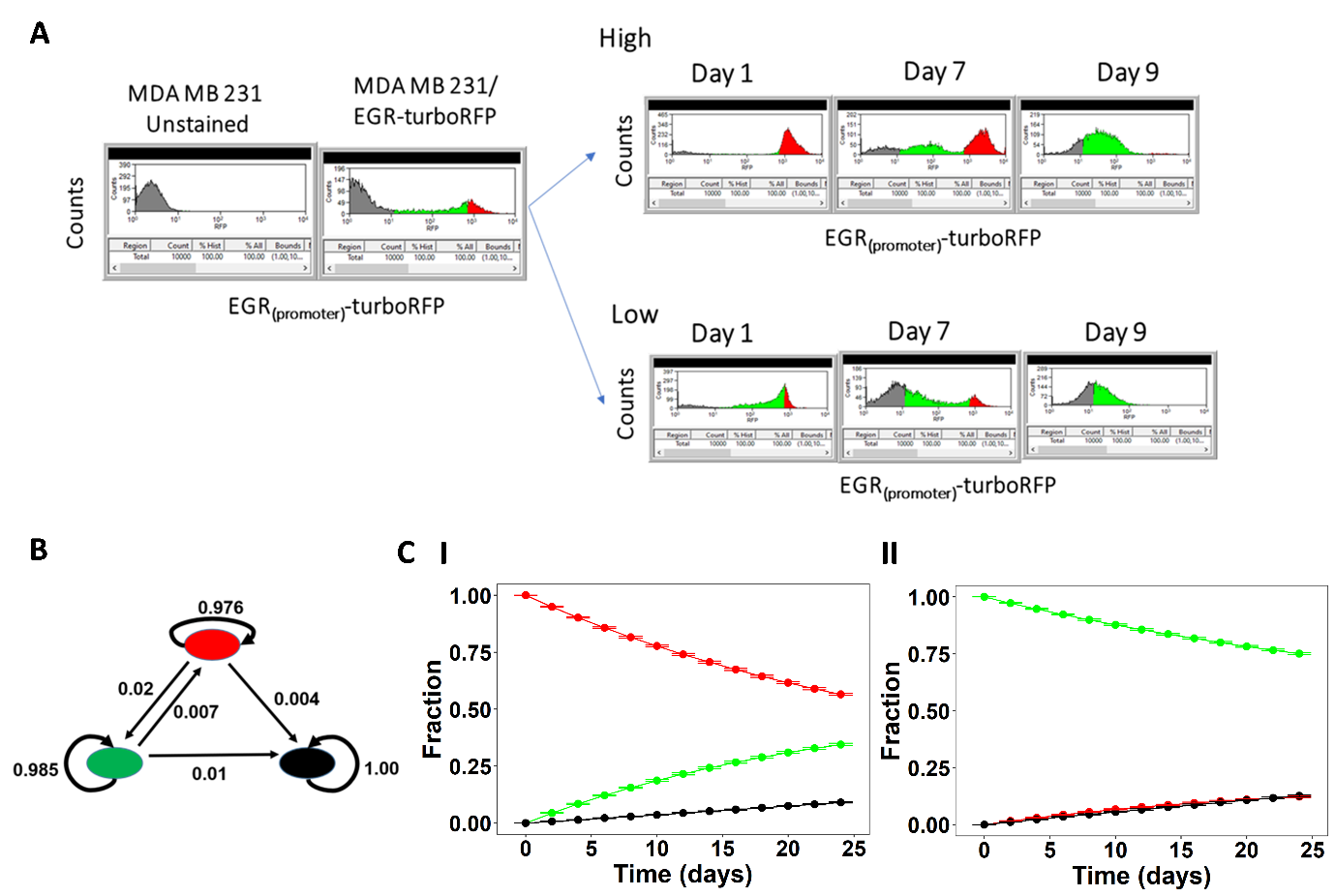


**Figure S9: A)** Experimental validation of AMPK-Akt feedback loop using MDA-MB-231 EGR-Turbo RFP cell lines sorted for high and low population using FACS, Red population corresponds to Low AMPK, High ERK and High Akt and Green population corresponds to High AMPK, Low Erk and Low Akt. Histograms show the conversion of population from High to low and vice-versa after 1,7 and 9 days when started with pure High and Low populations.

**B)** Transition rates inferred based on experimental data.  **C)** Plots show the evolution of populations initialized with pure High (left) and Low (right) over time produced using Markov chain simulations.

**Supplementary table legends**

**Table S1:**Shows the literature reported values of parameter values (references given below)

1. Connolly, N.M.C.; D’Orsi, B.; Monsefi, N.; Huber, H.J.; Prehn, J.H.M. Computational Analysis of AMPK-Mediated Neuroprotection Suggests Acute Excitotoxic Bioenergetics and Glucose Dynamics Are Regulated by a Minimal Set of Critical Reactions. *PLoS One* **2016**, *11*, e0148326, doi:10.1371/journal.pone.0148326.

2. Adi, Y.A.; Adi-Kusumo, F.; Aryati, L.; Hardianti, M.S. A Dynamic Model of PI3K/AKT Pathways in Acute Myeloid Leukemia. *J. Appl. Math.* **2018**, *2018*, 1–9, doi:10.1155/2018/2983138.

3. Holczer, M.; Hajdú, B.; Lőrincz, T.; Szarka, A.; Bánhegyi, G.; Kapuy, O. A Double Negative Feedback Loop between mTORC1 and AMPK Kinases Guarantees Precise Autophagy Induction upon Cellular Stress. *Int. J. Mol. Sci.* **2019**, *20*, 5543, doi:10.3390/ijms20225543.

4. Hu, H.; Goltsov, A.; Bown, J.L.; Sims, A.H.; Langdon, S.P.; Harrison, D.J.; Faratian, D. Feedforward and feedback regulation of the MAPK and PI3K oscillatory circuit in breast cancer. *Cell. Signal.* **2013**, *25*, 26–32, doi:10.1016/j.cellsig.2012.09.014.

5. Liu, B.; Oltvai, Z.N.; Bayır, H.; Silverman, G.A.; Pak, S.C.; Perlmutter, D.H.; Bahar, I. Quantitative assessment of cell fate decision between autophagy and apoptosis. *Sci. Rep.* **2017**, *7*, 17605, doi:10.1038/s41598-017-18001-w.

6. Coccimiglio, I.F.; Clarke, D.C. ADP is the dominant controller of AMP-activated protein kinase activity dynamics in skeletal muscle during exercise. *PLOS Comput. Biol.* **2020**, *16*, e1008079, doi:10.1371/journal.pcbi.1008079.

7. HARDIE, D.G.; SALT, I.P.; HAWLEY, S.A.; DAVIES, S.P. AMP-activated protein kinase: an ultrasensitive system for monitoring cellular energy charge. *Biochem. J.* **1999**, *338*, 717, doi:10.1042/0264-6021:3380717.

| **Parameter** | **Equation/Description** | **Value in units** | **Ref** |
| --- | --- | --- | --- |
| Kac_AMPK | AMP-mediated AMPK phosphorylation AMPK +AMP -> pAMPK +AMP | 1.3 nM^-1^ S^-1^ | [1] |
| Kdac_AMPK | Dephosphorylation of pAMPK pAMPK +ATP -> AMPK +ATP | 20*10^-3^ nM^-1^ S^-1^ | [1] |
| Kac_AKT | Rate constant of AKT phosphorylation | 1 – 20 min^-1^ | [2] |
| Kdac_AKT | Rate constant of AKTp dephosphorylation by PP2A | 0.36 – 13.5 uM min^-1^ | [2] |
|  | Michaelis constant of AKT phosphorylation | 0.1 uM | [2] |
|  | Michaelis constant of AKTp dephosphorylation | 0.08 – 0.4 uM | [2] |
| Kac_AMPK | Phosphorylation of AMPK | 0.35 - 0.5 min^-1^ | [3] |
| Kdac_AMPK | Dephosphorylation of pAMPK | 0.1 - 0.5 min^-1^ | [3] |
| Kac_AKT | Phosphorylation of AKT | 1.5*10^4^ nM min^-1^ | [4] |
| Kdac_AKT | Dephosphorylation of pAKT | 45 min^-1^ | [4] |
| Kac_AMPK | Phosphorylation of AMPK by Nstress | 1.64*10^4^ nM min^-1^  [0, 0.1] | [5] |
| Kdac_AMPK | Dephosphorylation of pAMPK | 0.0159 min^-1^   [0, 1] | [5] |
| Kdac_AMPK | Dephosphorylation of pAMPK by ULK1 | 1.64*10^4^ nM min^-1^ [0, 1] | [5] |
| Kac_AMPK | Phosphorylation of AMPK by CaMKKBeta | 1.65*10^4^ nM min^-1^ [0, 0.01] | [5] |
| Kac_AKT | Phosphorylation of AKT by PI3K | 0.0189 nM min^-1^ [0, 0.01] | [5] |
| Kdac_AMPK | Dephosphorylation of pAMPK by p53c | 1.57*10^4^ nM min^-1^ [0, 1] | [5] |
| Kdac_AKT | Dephosphorylation of pAKT | 0.0176 min^-1^ [0, 0.01] | [5] |
| Kac_AMPK | Phosphorylation of AMPK | 5*10^-3^ mM S^-1^ | [6] |
| Kac_AMPK | Phosphorylation of AMPK-ATP | 5*10^-3^ mM S^-1^ | [6] |
| Kac_AMPK | Phosphorylation of AMPK-ADP | 7.5*10^-3^ mM S^-1^ | [6] |
| Kac_AMPK | Phosphorylation of AMPK-AMP | 2*10^-2^ mM S^-1^ | [6] |
| Kdac_AMPK | Dephosphorylation of pAMPK | 1*10^-2^ mM S^-1^ | [6] |
| Kdac_AMPK | Dephosphorylation of pAMPK-ATP | 1*10^-2^ mM S^-1^ | [6] |
| Kdac_AMPK | Dephosphorylation of pAMPK-ADP | 1*10^-3^ mM S^-1^ | [6] |
| Kdac_AMPK | Dephosphorylation of pAMPK-AMP | 1*10^-4^ mM S^-1^ | [6] |
|  | Michaelis constant of pAMPK dephosphorylation | 6.7*10-2 mM | [6] |
|  | Michaelis constant of AMPK phosphorylation | 1.4 mM | [6] |
|  | Hill coefficient for AMPK activation | 2.5 | [7] |

**Table S2:** Shows Pearson's correlation values and corresponding p values for the comparisons between AMPK, Akt, PHLPP2 and PP2Cα for three replicates.

| **node1** | **node2** | **R (Mean)** | **R (SD)** | **p value** |
| --- | --- | --- | --- | --- |
| AMPK | AKT | -0.3137 | 0.0032 | <0.00001 |
| AMPK | PHLPP2 | 0.5352 | 0.0126 | <0.00001 |
| AMPK | PP2Ca | -0.5306 | 0.0027 | <0.00001 |
| AKT | PHLPP2 | -0.5224 | 0.0067 | <0.00001 |
| AKT | PP2Ca | 0.5330 | 0.0065 | <0.00001 |
| PHLPP2 | PP2Ca | -0.4387 | 0.0138 | <0.00001 |

**Table S3:**Shows the 10 representative parameter sets used in the study. (Separate excel sheet)

**Table S4 :** Shows the Pearson’s correlation values and p values between AMPK (pT172) and Akt (pT308 and pS473) for 32 different the cancer genome atlas (TCGA) cancer cohorts.

| **Cancer name** | **n** | **R for AMPKpT172, AKTpT308** | **p value of AMPKpT172, AKTpT308** | **R for AMPKpT172, AKTpS473** | **p value of AMPKpT172, AKTpS473** |
| --- | --- | --- | --- | --- | --- |
| Pheochromocytoma and Paraganglioma | 80 | 0.50195 | <1.00E-05 | 0.45915 | 2.00E-05 |
| Testicular Germ Cell Tumors | 118 | 0.29808 | 0.00104 | 0.37963 | 2.00E-05 |
| Lung adenocarcinoma | 362 | 0.23999 | <1.00E-05 | -0.14577 | 0.00546 |
| Kidney Chromophobe | 63 | 0.23292 | 0.0662 | 0.45348 | 0.00019 |
| Adrenocortical carcinoma | 46 | 0.23264 | 0.11975 | 0.10736 | 0.4776 |
| Prostate adenocarcinoma | 351 | 0.22495 | 2.00E-05 | 0.11112 | 0.03744 |
| Uterine Carcinosarcoma | 48 | 0.22418 | 0.12557 | 0.10114 | 0.49395 |
| Glioblastoma multiforme | 205 | 0.21269 | 0.0022 | 0.16164 | 0.02059 |
| Kidney renal papillary cell carcinoma | 208 | 0.204 | 0.00312 | -0.18188 | 0.00856 |
| Kidney renal clear cell carcinoma | 445 | 0.18318 | 1.00E-04 | -0.23115 | <1.00E-05 |
| Uterine Corpus Endometrial Carcinoma | 404 | 0.13507 | 0.00655 | 0.03462 | 0.48778 |
| Brain Lower Grade Glioma | 427 | 0.1236 | 0.01057 | 0.13098 | 0.00672 |
| Liver hepatocellular carcinoma | 184 | 0.06849 | 0.35562 | -0.15592 | 0.03455 |
| Cervical squamous cell carcinoma and endocervical adenocarcinoma | 171 | 0.05934 | 0.44073 | -0.12319 | 0.10845 |
| Head and Neck squamous cell carcinoma | 346 | 0.05196 | 0.33517 | -0.08316 | 0.12262 |
| Pancreatic adenocarcinoma | 105 | 0.02497 | 0.8004 | -0.32462 | 0.00073 |
| Bladder Urothelial Carcinoma | 344 | -0.00941 | 0.86195 | -0.21704 | 5.00E-05 |
| Esophageal carcinoma | 126 | -0.01861 | 0.83617 | -0.13023 | 0.1461 |
| Mesothelioma | 61 | -0.04037 | 0.7574 | -0.10196 | 0.43428 |
| Rectum adenocarcinoma | 130 | -0.08501 | 0.33624 | -0.4094 | <1.00E-05 |
| Lung squamous cell carcinoma | 325 | -0.08618 | 0.12103 | -0.22375 | 5.00E-05 |
| Stomach adenocarcinoma | 392 | -0.09921 | 0.04966 | -0.24172 | <1.00E-05 |
| Breast invasive carcinoma | 874 | -0.09947 | 0.00324 | -0.19939 | <1.00E-05 |
| Thyroid carcinoma | 372 | -0.10105 | 0.0515 | -0.23196 | 1.00E-05 |
| Colon adenocarcinoma | 357 | -0.11032 | 0.03721 | -0.27963 | <1.00E-05 |
| Skin Cutaneous Melanoma | 353 | -0.13683 | 0.01006 | -0.28625 | <1.00E-05 |
| Lymphoid Neoplasm Diffuse Large B-cell Lymphoma | 33 | -0.19445 | 0.27822 | -0.24534 | 0.16877 |
| Thymoma | 90 | -0.21226 | 0.0446 | -0.28325 | 0.00683 |
| Ovarian serous cystadenocarcinoma | 411 | -0.26948 | <1.00E-05 | -0.2995 | <1.00E-05 |
| Sarcoma | 221 | -0.37101 | <1.00E-05 | -0.4519 | <1.00E-05 |
| Cholangio carcinoma | 30 | -0.51375 | 0.00369 | -0.6893 | 3.00E-05 |
| Uveal Melanoma | 12 | -0.52671 | 0.07851 | -0.64711 | 0.02293 |
